# Physical Functional Limitations among Aboriginal and Non-Aboriginal Older Adults: Associations with Socio-Demographic Factors and Health

**DOI:** 10.1371/journal.pone.0139364

**Published:** 2015-09-30

**Authors:** Lina Gubhaju, Emily Banks, Rona MacNiven, Bridgette J. McNamara, Grace Joshy, Adrian Bauman, Sandra J. Eades

**Affiliations:** 1 Aboriginal Health, Baker IDI Heart and Diabetes Institute, 99 Commercial Road, Melbourne, 3004, Victoria, Australia; 2 Chronic Disease Epidemiology, National Centre for Epidemiology and Population Health, The Australian National University, Acton, 2601, Australian Capital Territory, Australia; 3 Prevention Research Collaboration, Sydney School of Public Health, The University of Sydney, Level 6 The Hub, The Charles Perkins Centre (D17), Sydney, 2006, New South Wales, Australia; Iran University of Medical Sciences, ISLAMIC REPUBLIC OF IRAN

## Abstract

**Background:**

Australian Aboriginal people are disproportionately affected by physical disability; the reasons for this are unclear. This study aimed to quantify associations between severe physical functional limitations and socio-demographic and health-related factors among older Aboriginal and non-Aboriginal adults.

**Methods:**

Questionnaire data from 1,563 Aboriginal and 226,802 non-Aboriginal participants aged ≥45 years from the Sax Institute’s 45 and Up Study (New South Wales, Australia) were used to calculate age- and sex-adjusted prevalence ratios (aPRs) for severe limitation [MOS-PF score <60] according to socio-demographic and health-related factors.

**Results:**

Overall, 26% (410/1563) of Aboriginal participants and 13% (29,569/226,802) of non-Aboriginal participants had severe limitations (aPR 2.8, 95%CI 2.5–3.0). In both Aboriginal and non-Aboriginal participants, severe limitation was significantly associated with: being ≥70 vs <70 years old (aPRs 1.8, 1.3–2.4 and 5.3, 5.0–5.5, within Aboriginal and non-Aboriginal participants, respectively), none vs tertiary educational qualifications (aPRs 2.4, 1.7–3.3 and 3.1, 3.0–3.2), lower vs higher income (aPRs 6.6, 4.2–10.5 and 5.5, 5.2–5.8), current vs never-smoking (aPRs 2.0, 1.6–2.5 and 2.2, 2.1–2.3), obese vs normal weight (aPRs 1.7, 1.3–2.2 and 2.7, 2.7–2.8) and sitting for ≥7 vs <7 hours/day (aPRs 1.6, 1.2–2.0 and 1.6, 1.6–1.7). Severe limitations increased with increasing ill-health, with aPRs rising to 5–6 for ≥5 versus no chronic conditions. It was significantly higher in those with few vs many social contacts (aPRs 1.7, 1.4–2.0 and 1.4, 1.4–1.4) and with very high vs low psychological distress (aPRs 4.4, 3.6–5.4 and 5.7, 5.5–5.9).

**Conclusions:**

Although the prevalence of severe physical limitation among Aboriginal people in this study is around three-fold that of non-Aboriginal people, the factors related to it are similar, indicating that Aboriginal people have higher levels of risk factors for and consequences of severe limitations. Effective management of chronic disease and reducing the prevalence of obesity and smoking are important areas for attention.

## Introduction

Australian Aboriginal adults have an average life expectancy approximately 10 years less than non-Aboriginal Australians [1] and have greater levels of ill-health at all stages of life. Colonisation of Australia has had a profound influence on the social, emotional and physical health of Australian Aboriginal people. It is likely that the consequent disempowerment and dramatic shift in diet and lifestyle have played a major role in the deteriorating physical and emotional wellbeing of generations of Aboriginal people [2].

Over the years, although there have been some important improvements in the health of Australian Aboriginal people, little change in the high prevalence of chronic disease and disability has occurred [3]. The proportion of 45–64 year old people living with severe disability requiring assistance with core activities such as mobility, self-care or communication among Aboriginal Australians is estimated to be almost three times that of non-Aboriginal Australians [4]. Physical disability is the most common type of disability among Aboriginal people, affecting 82% of those with a severe/profound disability [4]. It has been hypothesised that the observed levels of severe physical disability among Aboriginal people are largely due to the complications resulting from chronic diseases such as diabetes, heart disease and chronic kidney disease, rates of which are significantly higher among Aboriginal people [4, 5].

Currently, population level data on the prevalence of chronic disease and disability is the major source of information on physical functioning among Aboriginal people. Investigation of physical functional limitations among Aboriginal and non-Aboriginal older adults and an understanding of its relationship to socio-demographic factors and health indicators is important in targeting appropriate types of support to those in greatest need, and hence should contribute to “closing the gap” in health outcomes between Aboriginal and non-Aboriginal Australians. However, there is little direct evidence of the factors associated with physical functional limitations in Aboriginal Australians, nor are direct comparisons of how these relationships compare with those in non-Aboriginal Australians. The aim of this study was to assess the relationship between severe physical functional limitations and a range of socio-demographic, health and psychosocial factors and chronic disease among Aboriginal and non-Aboriginal people. This is in keeping with the ‘differential vulnerability hypothesis’, which suggests that the factors associated with physical functional limitations differ among Aboriginal people compared to non-Aboriginal people.

## Materials and Methods

### Participant recruitment

The Sax Institute's 45 and Up Study is a large prospective cohort study of people aged 45 years and older [6] residing in New South Wales (NSW), Australia, which includes periodic health questionnaires and large-scale data linkage. Potential study participants were randomly selected from the Medicare Australia database, with oversampling in regional areas and of those aged 80 years and older. Residents in remote areas were completely enumerated. Baseline self-administered postal questionnaires were distributed from 1 January 2006 to 31 December 2008. Joining the study comprised of completing the baseline questionnaire and providing written informed consent for follow up through repeat questionnaires and linkage of participant data to health-related datasets. Further information about the 45 and Up Study can be found at https://www.saxinstitute.org.au/our-work/45-up-study.

### Ethical Approval

Ethical approval of the 45 and Up Study as a whole was granted by the University of New South Wales Human Research Ethics Committee. Ethical approval for the current study has also been received from the Aboriginal Health and Medical Research Council of NSW (Reference 912/13). Written informed consent was obtained from all participants of this study.

### Data collection

The analyses described in this paper used data collected in the baseline postal questionnaire of the 45 and Up Study, apart from remoteness of residence (see below). Details of the variables collected and a summary of the characteristics of Aboriginal and non-Aboriginal participants of the study have been previously published [7].

Aboriginal origin was determined by self-identification. The questionnaire contained the item “Are you of Aboriginal or Torres Strait Islander origin?” Participants who checked the boxes “Yes, Aboriginal” or “Yes, Torres Strait Islander” were included as Aboriginal and/or Torres Strait Islander. Data on Aboriginal and Torres Strait Islander participants have been combined due to the small number of participants who identified as Torres Strait Islander. The term ‘Aboriginal’ refers to both Aboriginal and/or Torres Strait Islander participants in keeping with advice from the Aboriginal Health and Medical Research Council of New South Wales.

**Fig 1:**
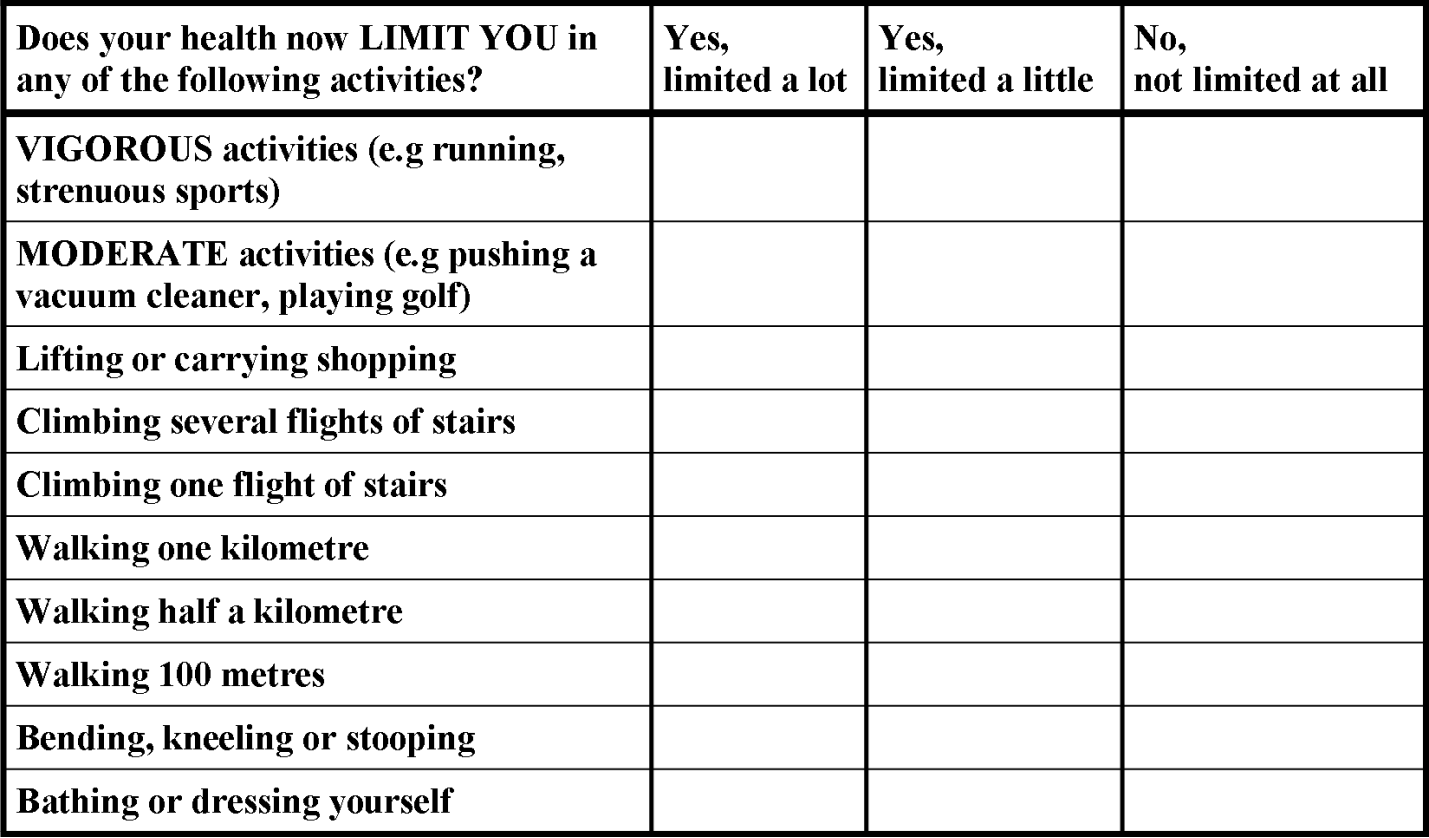
Questions included in the Medical Outcomes Study–Physical Functioning (MOS-PF) scale.

The degree of physical functional limitation was determined using the Medical Outcomes Study–Physical Functioning (MOS-PF) scale [8, 9] that asks participants 10 questions based on whether their health limits them in performing daily activities to vigorous activities (Fig 1). Participants were given a choice of three responses for each question with a score allocated for each response: 1) Yes, limited a lot (score = 1) 2) Yes, limited a little (score = 2) and 3) No, not limited at all (score = 3). Participants could score a minimum of 10 points and a maximum of 30 points which were then re-scaled to a score between 0–100 (10 = 0 and 30 = 100) with higher scores indicative of better physical functioning. Scores from this scale were categorized as follows: no limitation (score of 100); minor limitation (score 90–99); moderate limitation (60–89); and severe limitation (score 0–59).

The Accessibility Remoteness Index of Australia Plus (ARIA+) score [10] and the Index of Relative Socio-economic Disadvantage (IRSD) [11] were derived for each participant’s postcode of residence at the time of recruitment as recorded by Medicare Australia. Participants were grouped into quintiles of the IRSD score, with quintile 1 being the most disadvantaged, and quintile 5 the least disadvantaged. The ARIA+ score was used to identify participant's place of residence, categorized as: 'Major City', Inner Regional', 'Outer Regional', 'Remote' and 'Very Remote.' Other socio-demographic information included: age, sex, marital status, highest formal educational qualification, household annual pre-tax income and current employment status. Educational qualifications were categorised as follows: None (No school certificate or other qualification), High School (School or intermediate certificate/Higher school or leaving certificate), Technical (Trade or apprenticeship/Certificate or diploma) and University degree or higher.

Variables related to health behaviours included smoking, alcohol consumption, body mass index (BMI), screen time, hours spent sitting, physical activity and diet. Self-reported weight and height measurements were used to calculate participant’s BMI, as their weight in kilograms divided by the square of their height in metres (kg/m^2^). BMI was categorized according to the World Health Organization (WHO) criteria as underweight (<18.5kg/m^2^), normal weight (18.5 kg/m^2^-24.99 kg/m^2^), overweight (25.0–29.99 kg/m^2^), obese class I (30.0–34.99 kg/m^2^), obese class II (35.0–39.99) and obese class III (≥ 40.0 kg/m^2^) [12]. Participants’ overall level of physical activity was classified according to their responses to questions on the number of weekly sessions (of any duration) of moderate and vigorous physical activity and episodes of walking for longer than 10 min, using items from the validated Australian Institute of Health and Welfare’s (AIHW) Active Australia questionnaire [13]. A weighted weekly average number of sessions were calculated for each participant by adding the total number of sessions, with vigorous activity sessions receiving twice the weighting of moderate activity or walking sessions. Physical activity was classified as either ‘sufficient’ (150 min of physical activity in 5 or more sessions a week) or ‘insufficient’ (greater than 1 but less than or equal to 149 min), based on the guidelines from the AIHW [13]. Sedentary time was assessed based on 'screen time' which was the number of hours spent per day watching television or using the computer and ‘sitting time’ which was the number of hours per day spent sitting. Fruit and vegetable (including both raw and cooked vegetables) intake was assessed as servings per day and classified as adequate (≥ 2 servings of fruit and ≥ 5 servings of vegetables per day) or inadequate (less than these amounts) according to the National Health and Medical Research Council guidelines [14].

The Kessler-10 (K-10) scale was used to measure psychological distress [15, 16]. The scale contains a series of ten questions related to signs and symptoms of distress in the past 4 weeks with response options of “none of the time”, “a little of the time”, “some of the time”, “most of the time”, or “all of the time”. Kessler-10 scores were classified into 4 groups: low psychological distress (score 10–15), moderate psychological distress (score 16–21), high psychological distress (score 22–29) and very high psychological distress (score 30 or higher). Self-rated health and quality of life were categorised into the following: Excellent/very good, good/fair and poor. In order to determine the level of social support provided by close contacts, participants were asked “How many people outside your home, but within 1 hour of travel, do you feel you can depend on or you feel very close to?” Based on the responses the social support variable was categorised as follows: none, 1–3 people, 4–6 people and 7 or more people. Social interaction was also measured with the questions, “How many times in the last week did you spend time with friends or family who do not live with you?” and “How many times in the last week did you go to meetings of social clubs, religious groups or other groups you belong to?” These variables were categorised as follows: none, 1–2 times, 3–4 times and 5 or more times.

Prevalence of chronic diseases was assessed based on the participant’s response to the questions “Has the doctor ever told you that you have…” followed by a list of conditions that the participant could select. Number of chronic conditions were categorised as: 0, 1–2 conditions, 3–4 conditions, 5–6 conditions and 7 or more conditions.

### Statistical analysis

To assess the internal consistency of the 10 items included in the MOS-PF scale, Cronbach's alpha coefficients were assessed, with a criterion of 0.7 used to define adequate internal consistency. To assess the factor structure of the MOS-PF scale, an exploratory factor analyses was undertaken in the Aboriginal and non-Aboriginal participants separately (PROC FACTOR). The number of factors to retain in the final analysis was determined by examining eigenvalues (>1.0) [17].

Modified Poisson regression which combines a log Poisson regression model with robust variance estimation [18] was used to obtain age- and sex- adjusted prevalence ratios (aPR) for severe physical functional limitations (MOS-PF score of 0–59) for a range of socio-demographic and health indicators for Aboriginal and non-Aboriginal participants separately. A modified Poisson regression model was chosen over other log binomial regression and logistic regression models since it has been previously reported that use of the binomial regression model have limitations such as convergence difficulties [19]. Furthermore, a logistic regression model is known to produce odds ratios which are overestimated especially when the outcome is common [19]. Effect modification of the relationship between each specific factor (e.g sex) and severe physical functional limitation by Aboriginal status was assessed separately by comparing the model with and without the interaction term.

The MOS-PF score was also analysed as a continuous variable. Given the skewed distribution of the MOS-PF scores (as shown by the Kolmogorov-Smirnov test [P<0.01]), group medians and interquartile ranges are reported along with means and standard deviations. Non-parametric Kruskal-Wallis statistical tests were utilised to examine significant variation in median scores within categories of the exposure variables (for example to examine variation in MOS-PF scores by age groups, median scores within age group categories [45–49 years, 50–59 years, 60–69 years, 70+ years] were compared). All statistical analyses were undertaken using SAS software version 9.3 (SAS Institute Inc, Cary, NC, USA). Statistical significance was accepted at the P<0.05 level.

### Inclusion/Exclusion criteria

Participants without a valid age or date of entry into the study or an invalid response to the question on Aboriginal origin (n = 4741), or without a valid MOS-PF score following the logical backfilling (n = 386 Aboriginal, n = 33614 non-Aboriginal) were excluded from the analysis.

## Results

The study population available for analyses included 1563 Aboriginal and 226802 non-Aboriginal participants. Baseline socio-demographic and health characteristics of these participants are given in Table 1. The proportion of participants in the younger age groups was higher among Aboriginal people compared to non-Aboriginal people.

**Table 1:** Characteristics of Aboriginal and non-Aboriginal participants of the 45 and Up study examined in the current study and overall MOS-PF score and level of physical functional limitations.

	Aboriginal	Non-Aboriginal
	(n = 1563)	(n = 226802)
	% (n)	% (n)
**Sex**		
Male	44 (690)	47 (107413)
Female	56 (873)	53 (119389)
**Age (years)**		
45–49	24 (373)	14 (31618)
50–59	45 (701)	35 (78927)
60–69	21 (336)	28 (63658)
≥70	10 (153)	23 (52599)
**Educational qualifications**		
None	27 (415)	10 (23314)
High school	29 (453)	31 (70858)
Technical (Trade/Diploma/Certificate)	27 (427)	33 (73930)
Uni or higher	15 (236)	25 (56134)
**Work Status**		
Paid work	36 (565)	33 (74416)
Home/Family	8 (118)	7 (15419)
Retired	20 (307)	35 (78304)
Disabled/sick	14 (214)	3 (7662)
Unemployed	4 (68)	1 (3110)
Other	18 (277)	21 (46771)
**Annual household income**		
<$20,000	31 (480)	18 (41624)
$20,000-$39,000	18 (279)	18 (40449)
$40,000-$69,000	16 (251)	19 (42617)
≥$70,000	17 (263)	26 (58808)
**Marital status**		
Married/Partnered	62 (974)	76 (171859)
Single	13 (197)	6 (12582)
Widowed	6 (98)	8 (17262)
Divorced/Separated	18 (277)	11 (23926)
**Smoking status**		
Never	40 (630)	56 (126539)
Former	37 (583)	37 (83640)
Current	22 (339)	7 (16034)
**Body mass index**		
Underweight (<18.5 kg/m^2^)	1 (21)	1 (2689)
Normal weight (18.5–24.99 kg/m^2^)	20 (317)	34 (78014)
Overweight (25.0 kg/m^2^-29.99m^2^)	33 (518)	37 (83939)
Obese (≥30 kg/m^2^)	34 (535)	21 (46526)
**Median (IQR) MOS-PF score**	85 (55–100)	95 (80–100)
**Level of physical functional limitation**		
None (100)	29 (451)	34 (77476)
Minor (90–99)	20 (316)	29 (65438)
Moderate (60–89)	25 (386)	24 (54319)
Severe (0–59)	26 (410)	13 (29569)

Percentages may not equal to 100 due to missing/invalid data

MOS-PF = Medical Outcomes Study Physical Functioning

The MOS-PF scale demonstrated very high levels of internal consistency among both the Aboriginal and non-Aboriginal group with Cronbach’s alphas of 0.98 and 0.99, respectively. Exploratory factor analysis showed a similar one-dimensional factor structure among Aboriginal and non-Aboriginal participants based on eigenvalues greater than one. Only one factor among both Aboriginal and non-Aboriginal participants showed eigenvalues greater than one (6.64 and 6.12, respectively). All ten items included on the MOS-PF scale were shown to have a moderate to high loading (>0.50) onto that single factor (S2 Table).

Median MOS-PF scores were lower among Aboriginal participants compared to non-Aboriginal participants (Table 1). Overall, 26% of Aboriginal participants and 13% of non-Aboriginal participants had scores consistent with severe limitation (Score <60). After adjusting for age and sex, the prevalence of severe limitation among Aboriginal people was around three times that of non-Aboriginal people (aPR: 2.8, 95% CI 2.5–3.0).

### Socio-demographic factors

**Fig 2:**
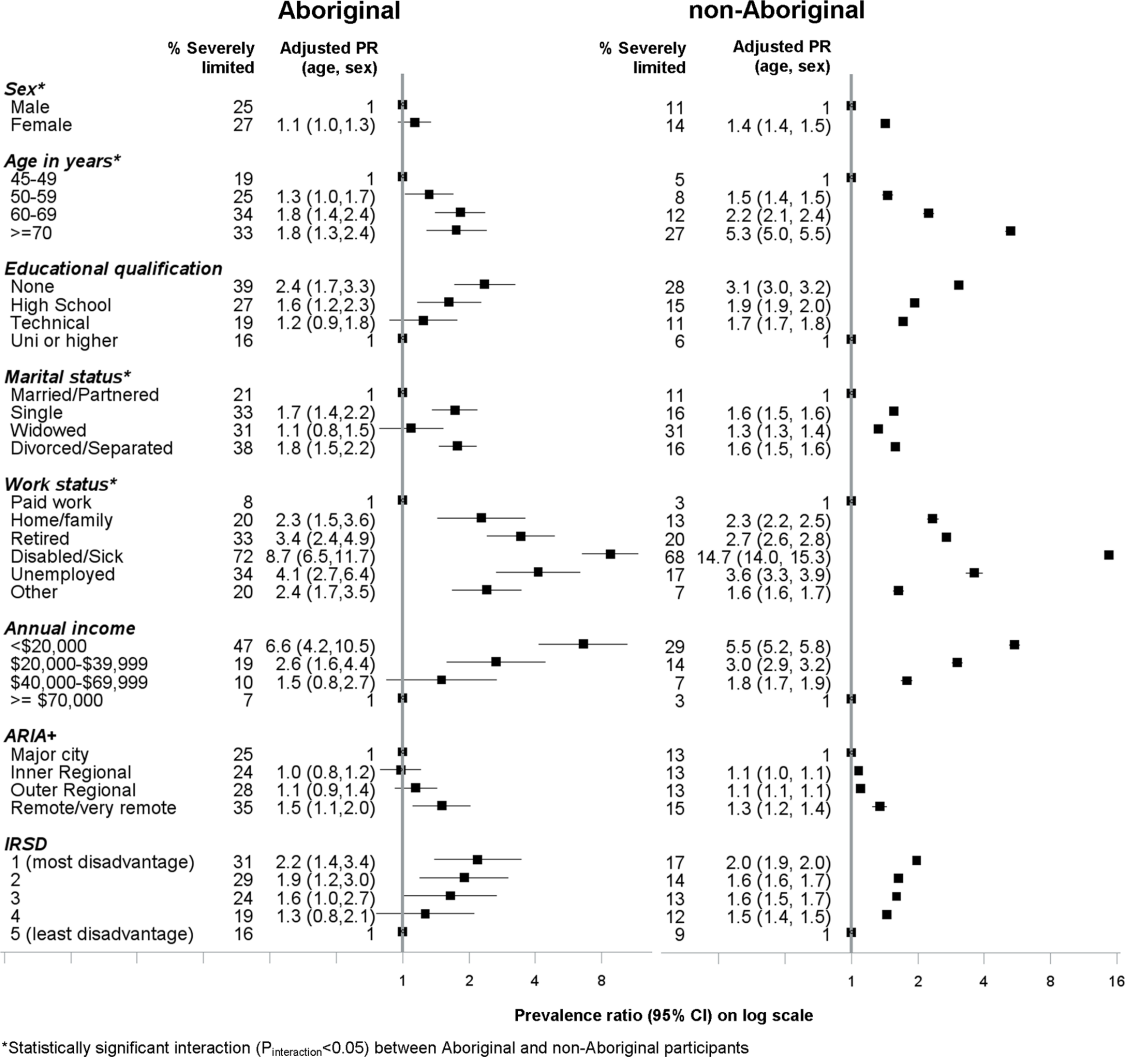
Association between *severe physical functional limitations* (MOS-PF score 0–59) and socio-demographic factors among Aboriginal and non-Aboriginal participants from the 45 and Up study.

In both Aboriginal and non-Aboriginal participants, the prevalence of severe limitation increased with increasing age (Fig 2), with a steeper gradient in the prevalence ratio among non-Aboriginal participants (P_interaction_<0.001), but similar differences in absolute prevalence according to age within the two groups. Overall, 19% of Aboriginal participants aged between 45–49 years were severely limited compared to 5% of non-Aboriginal participants.

The prevalence of severe limitation was generally greater in females compared to males. In both Aboriginal and non-Aboriginal participants, severe limitation was significantly higher among participants who had fewer educational qualifications, not married or partnered, not in paid employment, low income earners, and among those living in remote areas and areas with greater social disadvantage, compared to those without these characteristics (Fig 2). It is to be noted that among those in paid employment, the absolute prevalence of severe limitation was higher among Aboriginal participants (8%) versus non-Aboriginal participants (3%). Although the relationship between socio-demographic factors and severe limitation were generally similar between Aboriginal and non-Aboriginal participants, significant statistical interaction with Aboriginal status was found with age (P_interaction_<0.001), sex (P_interaction_ = 0.05), marital status (P_interaction_ <0.001) and work status (P_interaction_<0.001).

### Health behaviours

**Fig 3:**
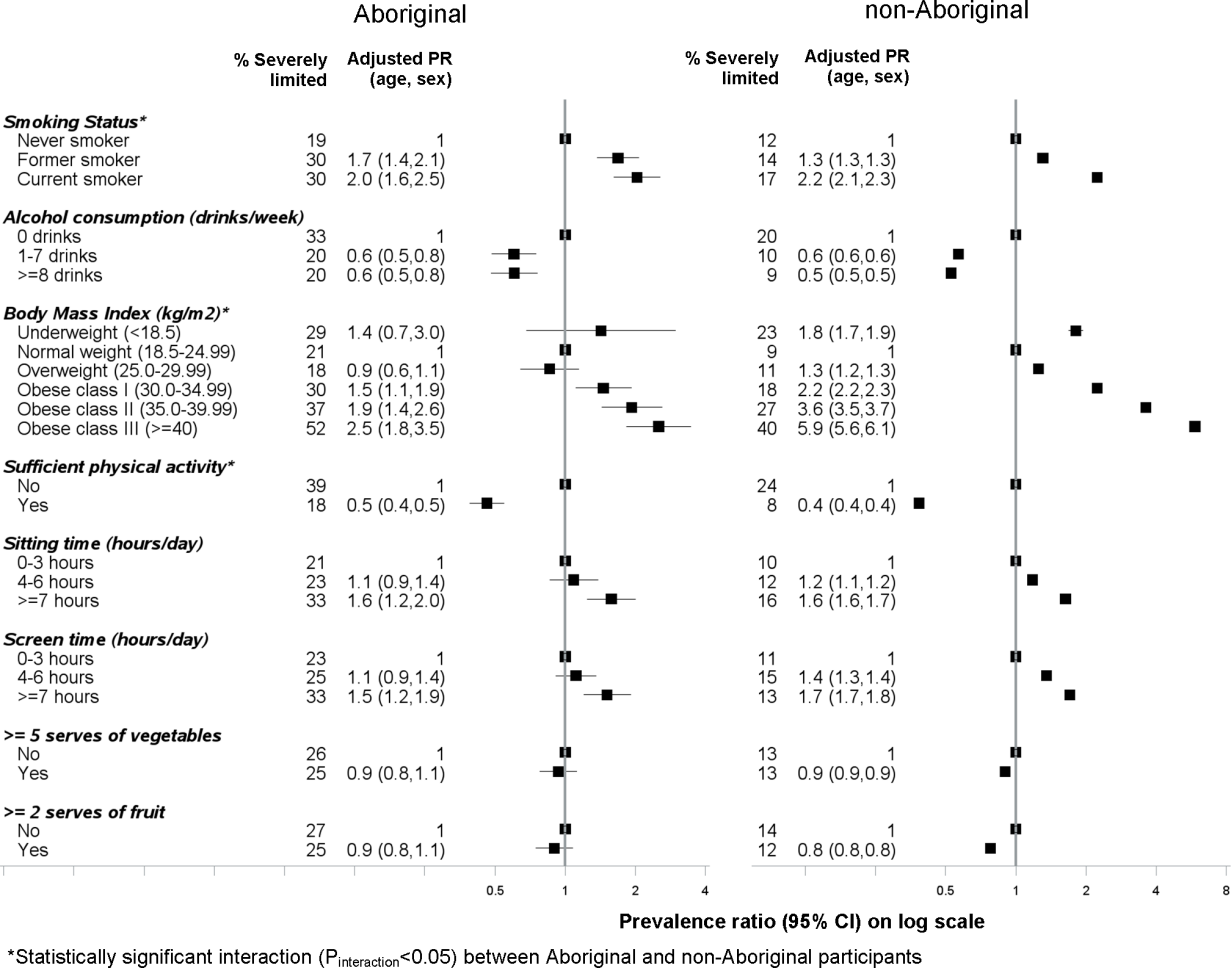
Association between *severe physical functional limitations* (MOS-PF score 0–59) and health behaviours among Aboriginal and non-Aboriginal participants from the 45 and Up study.

Among both Aboriginal and non-Aboriginal participants, those who were overweight or obese were more likely to be severely limited compared to those with normal weight (Fig 3). The prevalence of severe functional limitation also increased with increasing severity of obesity (class I–class II); with a steeper gradient in prevalence ratio among non-Aboriginal vs Aboriginal people (P_interaction_<0.001). The prevalence of severe functional limitation was greater among those with higher versus lower levels of sedentary time (screen time and sitting time) and lower (50%-60%) among those people who achieved the recommended levels of physical activity compared to those people who did not. Significant statistical interaction with Aboriginal status was found with meeting the physical activity recommendations (P_interaction_ = 0.02).

Among both Aboriginal and non-Aboriginal participants, current smokers had twice the prevalence of severe limitation compared to non-smokers. Among non-Aboriginal participants the absolute proportions of participants severely limited was lower among former smokers compared to current smokers (14% vs 17%); however, the proportion was similar among Aboriginal participants (30%). Furthermore, the absolute proportion of participants who were severely limited was 10% higher among Aboriginal current smokers compared to non-smokers whereas among non-Aboriginal participants, the difference in proportions was 5%. Significant statistical interaction with Aboriginal status was found with smoking status (P_interaction_ = 0.02). Participants who consumed more alcohol were less likely to be severely limited compared to those who were non-drinkers or consumed less than 1 drink/week.

### Psychosocial factors

**Fig 4:**
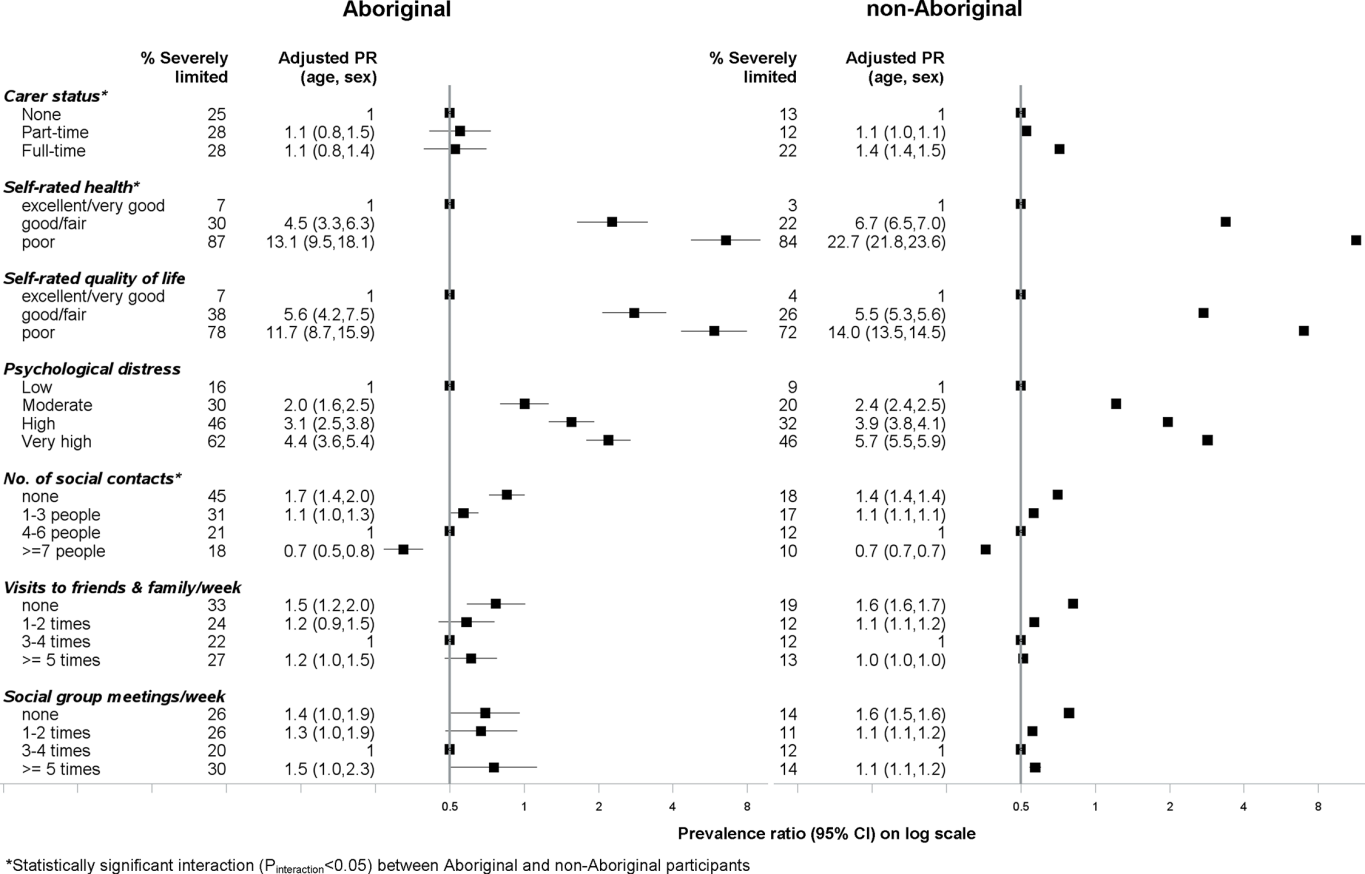
Association between *severe physical functional limitations* (MOS-PF Score 0–59) and psychosocial factors among Aboriginal and non-Aboriginal participants from the 45 and Up study.

Among Aboriginal and non-Aboriginal participants who rated their health as poor, 87% and 84%, respectively, had scores consistent with severe limitation (Fig 4). The prevalence of severe limitation increased with increasing levels of psychological distress among both Aboriginal and non-Aboriginal participants with prevalence ratios of 2.0 among those with moderate distress and as high as 5.7 among those with very high levels of distress. Aboriginal and non-Aboriginal participants with very high levels of psychological distress had scores consistent with severe limitations (62% and 46%, respectively).

Having no one to depend on (outside of home) was associated with a higher prevalence of severe limitation among Aboriginal and non-Aboriginal people, with a much higher absolute prevalence among Aboriginal people (45% vs 18%). Accordingly, prevalence ratios of 0.7 were found among both Aboriginal and non-Aboriginal people who responded having seven or more people who they could depend on compared to those with no social contacts. Prevalence of severe limitation was significantly higher among those who spent no time with friends or family who did not live with them compared to those that spent 3–4 times per week, among both Aboriginal and non-Aboriginal people. In terms of meetings of social clubs, religious groups and other groups, prevalence of severe limitation was high among those who attended none or only 1–2 meetings per week and those who attended many meetings (5 or more) per week compared to those attending 3–4 times per week. Furthermore, prevalence of severe limitation was higher among those participants who had full-time carer responsibilities compared to those who had no carer responsibilities. Significant statistical interaction with Aboriginal status was found with carer status (P_interaction_<0.001), social contacts (P_interaction_ = 0.03) and self-rated health (P_interaction_ = 0.03).

### Chronic diseases

**Fig 5:**
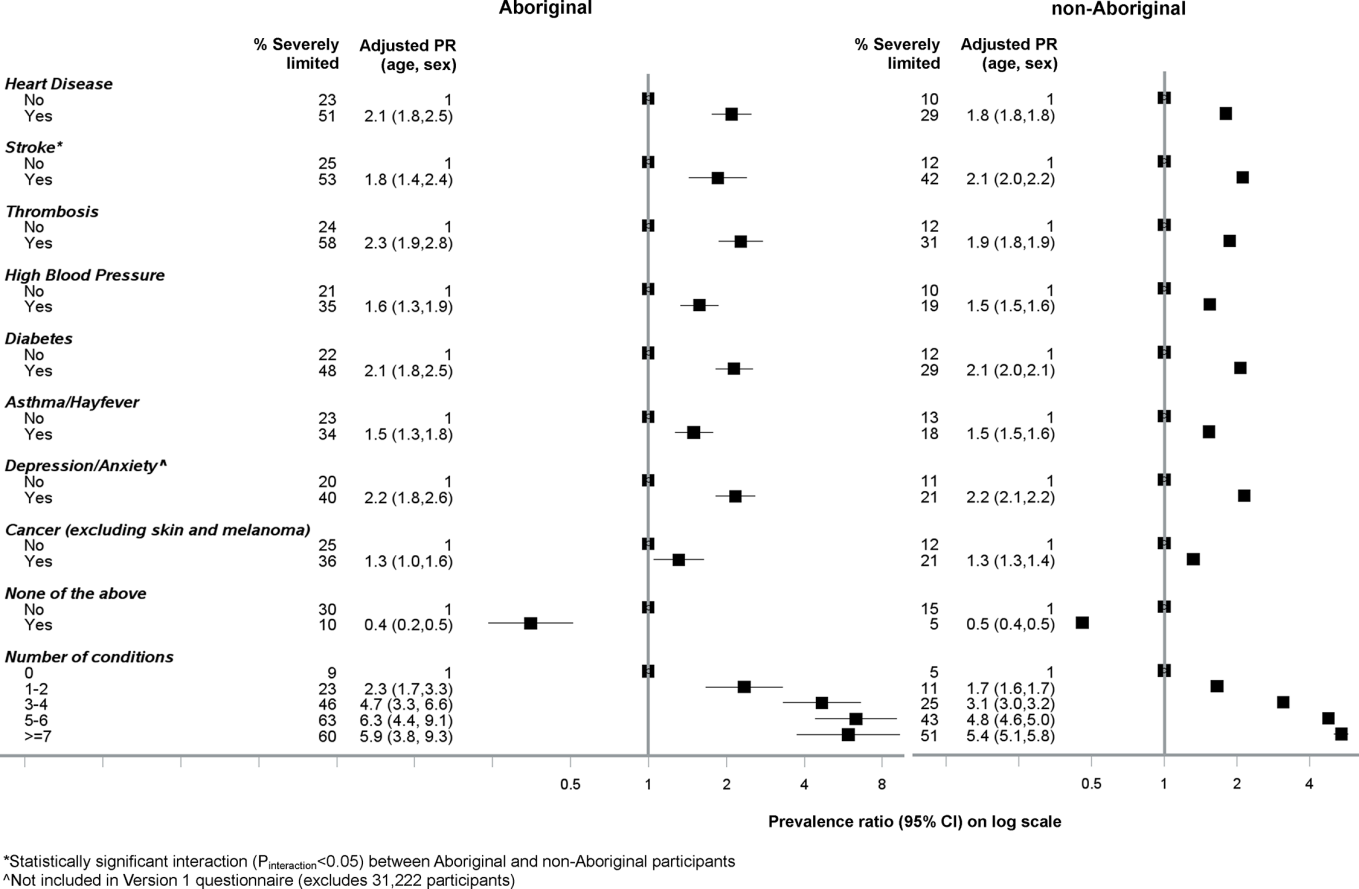
Association between *severe physical functional limitations* (MOS-PF score 0–59) and chronic disease among Aboriginal and non-Aboriginal participants from the 45 and Up study.

A higher prevalence of severe limitation was found among individuals diagnosed with a specific medical condition in comparison to those that did not have the condition. Absolute baseline prevalence of severe limitation was consistently high among Aboriginal people: approximately 20–25% of Aboriginal people who responded as not being diagnosed with the specific condition were severely limited. Among those who responded to not being diagnosed with *any* of the conditions listed, 10% of Aboriginal people were severely limited compared to 5% of non-Aboriginal people. Aboriginal and non-Aboriginal participants who had ever been told by a doctor that they had diabetes had 2.1 times the prevalence of severe limitation, respectively, compared to those that did not have diabetes (Fig 5). The prevalence of severe functional limitation among participants who had thrombosis, heart disease, depression/anxiety and stroke was also twice that of those without those conditions. Prevalence of severe limitation increased steadily with increasing number of chronic conditions; compared to those with no chronic conditions, a prevalence ratio of 2.3 (95% CI 1.7–3.3) was observed among Aboriginal participants with one to two conditions; rising to 5.9 (95% CI 3.8–9.3) among those with seven or more conditions. Significant statistical interaction with Aboriginal status was found for the relationship between severe limitation and stroke (P_interaction_ = 0.01).

### Differences in median MOS-PF score (S2 Table)

Differences in median MOS-PF score among Aboriginal and non-Aboriginal people according to socio-demographic factors, health behaviours, psychosocial factors and chronic disease and disability showed similar results to those from the prevalence ratios described above (S2 Table).

## Discussion

The prevalence of severe limitation among middle-aged and older Aboriginal people in this study was approximately three times that of non-Aboriginal people, such that over one-quarter of Aboriginal participants had severe physical limitations. Among both Aboriginal and non-Aboriginal participants, severe limitation was associated with older age, socio-economic disadvantage, being a former or current smoker, obesity, sedentary behaviour (screen time and sitting time), poor self-rated health and quality of life, high psychological distress and fewer social contacts. Prevalence of severe limitation also increased steadily with increasing number of chronic conditions. Although Aboriginal people had a consistently higher absolute prevalence of severe limitation, in general the factors relating to severe limitation were similar for Aboriginal and non-Aboriginal participants. This suggests that Aboriginal people may not have differential vulnerability to physical functional limitations, but experience a higher prevalence of the factors that are related to higher levels of physical disability in the population as a whole.

The prevalence of severe limitations increased with age among both Aboriginal and non-Aboriginal participants. However, the gradient of increasing physical limitation with increasing age was less steep for Aboriginal compared to non-Aboriginal participants, both in relative and absolute terms. A contributing factor is likely to be the very high prevalence of severe limitation among younger Aboriginal participants compared to their non-Aboriginal counterparts, with 19% of Aboriginal participants aged 45–49 having severe limitations; this may reflect the premature morbidity and earlier onset of chronic conditions among Aboriginal people [20, 21]. It has previously been reported that 84% of Aboriginal people who access disability support services are less than 50 years of age [22]. Appropriate management of risk factors (social disadvantage, health risk factors) and chronic disease associated with severe limitation, as identified in the current study, at a younger age may avoid further age-related impairment among Aboriginal people.

In accordance with previous findings, socio-economic disadvantage (low levels of formal education, non-paid work status and low annual household income) were important correlates of severe limitation [4, 23]. Our study has shown that the relationship between socio-demographic factors and severe limitations to be very similar between Aboriginal and non-Aboriginal people, suggesting socio-economic disadvantage to be a common factor on the causal pathway to physical limitations, as well as being a consequence of physical limitation/disability. However, the proportion severely limited was consistently higher among Aboriginal people in each category. In particular, 47% of Aboriginal participants with a low annual household income (<$20,000) were severely limited compared to 29% of non-Aboriginal participants. This finding is in accordance to previous literature that has shown the strong relationship between social disadvantage and ill health among Aboriginal people [24, 25]. Among people in paid employment or looking after their home/family, a higher absolute proportion of Aboriginal people were severely limited compared to non-Aboriginal people (8% vs 3% and 20% vs 13%, respectively). This suggests that, despite physical limitations, Aboriginal people persist with work and home duties, suggestive of resilience to physical ailments.

The prevalence of severe limitation was significantly higher among both former and current smokers compared to never smokers, particularly for Aboriginal participants. Smoking is a known risk factor for a number of chronic diseases and has previously been shown to be a significant contributor to the difference in the disability-adjusted life years between Aboriginal and non-Aboriginal people [21]. Although smoking rates in the general population have declined over recent years, 41% of Aboriginal people are still daily smokers [22] and 51% of Aboriginal people who responded as being severely disabled in a national survey have been daily smokers [26]. Therefore, the findings of this study further support existing efforts to reduce smoking among Aboriginal people, which is likely to be a key intervention for reducing the causes and consequences of disease and disability. Interestingly, the current study showed that both Aboriginal and non-Aboriginal participants who reported consuming more than 1 drink per week were less likely to have severe physical functional limitations compared to non-drinkers. This may be because those participants who suffer from poor physical health have been advised to abstain from alcohol; similar findings have been reported in previous health surveys whereby the prevalence of medium-to-high risk level drinking was lower among those with severe/profound disability compared to those with no disability [4].

The current study showed that prevalence of severe physical functional limitations increased with increasing BMI; importantly, we found a graded increase in the prevalence of severe limitation with increasing severity of obesity (class I–class III). Our findings are supported by a recent systematic review and meta-analysis that also demonstrated a graded increase in the risk of disability (limitations in activities of daily living) among overweight and obese relative to normal weight [27]. The strong association between obesity and chronic disease is also likely to be contributing to the relationship between obesity and physical functional limitations. The gradient of the relationship between severity of obesity and functional limitations was less steep among Aboriginal people; this may be due to the already high prevalence of functional limitations among those who were normal weight compared to their non-Aboriginal counterparts (21% vs 9%).

In agreement with previous reports [28–30], there was a significant association between prevalence and number of chronic diseases and severe physical functional limitations. It is important to note that baseline prevalence of severe limitation among Aboriginal people was high (20–25% among those that were not diagnosed with the specific medical condition) even though adjusted prevalence ratios for severe limitation were similar between Aboriginal and non-Aboriginal people. Furthermore, even among Aboriginal people who responded to having none of the conditions listed, 10 percent were still severely limited; double the prevalence among non-Aboriginal participants, which suggests an important contributory role of other factors and conditions.

The relationship between physical and psychological wellbeing has been explored in a number of recent studies [31–35]. In this study, there was a significant association between severe limitations and history of depression/anxiety and also a significant gradient in the prevalence of severe limitation among those with increasing levels of psychological distress in both Aboriginal and non-Aboriginal participants. Interestingly, a higher proportion of Aboriginal people who self-rated their health as excellent/very good were severely limited compared to non-Aboriginal people (7% vs. 3%) suggesting that Aboriginal people’s perception of their health may include factors other than their physical state.

It has recently been reported that Australians with disabilities were less likely to belong to networks and to have social support [36]. In the current study, people with severe physical limitations had generally lower levels of social contact compared to those with fewer limitations, as measured by number of social contacts, visits to family and friends and membership of social groups. It should be noted that the Duke Social Support Scale used in the 45 and Up Study focuses on social contacts outside the home, which may not capture social support within the household or family; such household and family support may be of greater importance to Aboriginal people.

This study has a number of strengths including the large population-based sample of Aboriginal people aged 45 years and older from New South Wales, which has the largest number of Aboriginal people in Australia. The MOS-PF scale is a well-validated tool that has been widely used in many population based studies and the cross-cultural validity of the whole Short Form-36 tool has also been assessed [37]. The current study further confirms that the uni-dimensional factor structure of the MOS-PF scale which was shown among both Aboriginal and non-Aboriginal people. To date, severe limitations have mainly been examined through the prevalence of chronic disease and disability; which does not clearly show the level of physical impairments among the population. Hence, use of the MOS-PF scale allows a more detailed examination of physical limitations among older adults.

There are also some limitations to this study which need to be acknowledged. The approximately 18% response rate for participation in the 45 and Up Study (which is in keeping with other studies of its kind) means there is likely to be a “healthy cohort effect” such that participants are likely to be healthier and less physically limited compared to the general population. However, this effect has been shown not to materially affect within-cohort comparisons, such as those presented here [38]. In this regard, high levels of severe limitation among these Aboriginal participants suggest that the true burden is likely to be even greater. Furthermore, given that the study was based on self-reported data, it is possible that the physical functional limitation score in Aboriginal and non-Aboriginal groups may have been affected by response style bias (socially desirable responding) [39] which has been shown to be more common among ethnic minorities [40] and therefore may have differed by Aboriginal status in the current study.The current analyses were based on the cross-sectional baseline survey data; therefore, temporal relationships between the risk factors and severe limitations could not be established. However, the longitudinal design of the overall 45 and Up Study will allow future analyses of temporal relationships and also an examination of the changes in physical functional limitations among study participants.

This study presents both absolute prevalence of severe physical functional limitation–to demonstrate absolute differences across various factors and between Aboriginal and non-Aboriginal participants–and prevalence ratios, to illustrate relative differences. In addition, effect modification with Aboriginal status on the relationships of factors with physical limitations has also been assessed. Therefore, it is important that all three measures are considered in interpreting the relationships of factors with physical impairment among Aboriginal and non-Aboriginal people. Due to the very high absolute prevalence of severe limitations among the Aboriginal participants, potential ceiling effects limit the magnitude of the prevalence ratios in Aboriginal participants to a greater extent than non-Aboriginal participants. For example, if the prevalence of severe limitation is 20% in the non-exposed group, it is not possible to have relative risks above 5. Similarly, when baseline prevalence is high, prevalence ratios may not appear particularly dramatic but absolute differences can be large. For example, for both Aboriginal and non-Aboriginal participants, those aged 60–69 years have around double the prevalence of severe physical limitations of those aged 45–49 years. However, the absolute difference between these groups is around 15% (19% vs 34%) in Aboriginal participants and 7% (5% vs 12%) in non-Aboriginal participants.

In terms of the relationship between functional limitations and chronic disease, conditions that participants selected from a list on the questionnaire were used in this study; this means that common conditions were captured, but other less common conditions that were listed as free text by participants were not included. Finally, this study focuses on the more proximal “upstream” factors relating to disability and functional limitations, including poverty, illness, and health behaviours. It does not capture the broader societal and cultural determinants of these factors, such as colonisation and its related consequences including disempowerment, racism and discrimination.

## Conclusions

Aboriginal people in the 45 and Up Study have a significantly greater burden from physical functional limitations compared to non-Aboriginal people. The relationships of socio-economic, health and psychosocial factors to severe limitation among Aboriginal and non-Aboriginal people were very similar. Taken together, these indicate that Aboriginal people have greater levels of risk factors for and consequences of severe physical limitation, and these occur at younger ages. The major role of ill health in disability highlights the importance of continuing efforts in chronic disease management and the need to address the smoking and obesity epidemics.

## Supporting Information

S1 Table Loading of the 10 items of the MOS-PF on the single factor retained by the exploratory factor analysis among Aboriginal and non-Aboriginal participants. (DOCX)

S2 Table Mean and median MOS-PF score in Aboriginal and non-Aboriginal participants stratified by groups according to socio-demographic factors, health behaviours, psychosocial factors and chronic diseases. (DOCX)
