# Supplementary material for: Physical Functional Limitations among Aboriginal and Non-Aboriginal Older Adults: Associations with Socio-Demographic Factors and Health
Source: PLoS One. 2015 Sep 30;10(9):e0139364. doi: 10.1371/journal.pone.0139364 (PMC4589378; doi:10.1371/journal.pone.0139364)
Supplement: S1 Table — (DOCX) [file pone.0139364.s001.docx]

| **Item on MOS-PF scale**  *Does your health now limit you in any of the following activities:* | **Aboriginal** | **Non-Aboriginal** |
| --- | --- | --- |
| Vigorous activities (e.g. running, strenuous sports) | 0.68 | 0.58 |
| Moderate activities (e.g. pushing a vacuum cleaner, playing golf) | 0.84 | 0.82 |
| Lifting or carrying shopping | 0.82 | 0.79 |
| Climbing several flights of stairs | 0.85 | 0.82 |
| Climbing one flight of stairs | 0.85 | 0.85 |
| Walking one kilometre | 0.90 | 0.88 |
| Walking half a kilometre | 0.89 | 0.88 |
| Walking 100 metres | 0.81 | 0.79 |
| Bending, kneeling or stooping | 0.76 | 0.71 |
| Bathing or dressing yourself | 0.70 | 0.63 |

S2 Table. Loadings of the 10 items of the MOS-PF on the single factor retained by the exploratory factor analysis among Aboriginal and non-Aboriginal participants
