# Supplementary material for: Physical Functional Limitations among Aboriginal and Non-Aboriginal Older Adults: Associations with Socio-Demographic Factors and Health
Source: PLoS One. 2015 Sep 30;10(9):e0139364. doi: 10.1371/journal.pone.0139364 (PMC4589378; doi:10.1371/journal.pone.0139364)
Supplement: S2 Table — (DOCX) [file pone.0139364.s002.docx]

S2 Table. Mean and median MOS-PF score in Aboriginal and non-Aboriginal participants stratified by groups according to socio-demographic factors, health risk factors, psychosocial factors and chronic diseases

|  |  | **Aboriginal** | | **Non-Aboriginal** | |
| --- | --- | --- | --- | --- | --- |
|  |  | **Mean ± SD** | **Median (IQR)** | **Mean ± SD** | **Median (IQR)** |
| **SOCIO-DEMOGRAPHIC FACTORS** | |  |  |  |  |
| Sex | Male  Female  *F/chi-squared statistic* | 75.3 ± 30.1  73.2 ± 29.2  *2.1^ns^* | 90 (60-100)  85 (55-100)  ***5.5**** | 84.9 ± 21.8  82.4 ± 23.9  ***625***** | 95 (80-100)  95 (75-100)  ***296.2***** |
| Age | 45-49  50-59  60-69  70+  *F/chi-squared statistic* | 80.2 ± 26.2  75.5 ± 29.6  67.3 ± 30.8  68.1 ± 31.2  ***14.1***** | 95 (70-100)  90 (60-100)  78 (45-95)  80 (45-95)  ***54.9***** | 91.6 ± 16.4  88.7 ± 18.7  84.0 ± 21.3  70.7 ± 28.2  ***9107***** | 100 (90-100)  95 (85-100)  90 (80-100)  80 (55-94)  ***30999***** |
| Marital status | Married/Partnered  Single  Widowed  Divorced/Separated  *F/chi-squared statistic* | 77.7 ± 28.1  69.4 ± 31.2  68.1 ± 31.2  67.1 ± 31.0  ***10.0***** | 90 (65-100)  80 (45-100)  75 (50-95)  80 (44-95)  ***43.3***** | 85.6 ± 21.0  81.5 ± 24.6  68.3 ± 30.4  81.2 ± 24.8  ***2424***** | 95 (80-100)  90 (75-100)  80 (45-95)  90 (75-100)  ***7287***** |
| Education | None  High School  Technical  Uni or higher  *F/chi-squared statistic* | 66.0 ± 31.8  73.5 ± 30.4  78.6 ± 26.7  82.0 ± 24.1  ***15.1***** | 75 (40-95)  89 (55-100)  90 (67-100)  95 (75-100)  ***51.2***** | 71.9 ± 30.2  81.7 ± 23.9  84.7 ± 21.5  89.8 ± 16.8  ***2937***** | 85 (50-95)  90 (75-100)  95 (80-100)  95 (90-100)  ***8805***** |
| Work status | Part/full-time  Unemployed  Retired  Disabled/sick  Home/family  Other  *F/chi-squared statistic* | 87.6 ± 18.3  70.2 ± 29.6  68.3 ± 30.3  40.1 ± 29.8  77.4 ± 23.6  79.3 ± 27.0  ***96.1***** | 95 (80-100)  80 (55-100)  80 (45-95)  35 (15-60)  85 (65-95)  90 (70-100)  ***366***** | 92.2 ± 13.2  80.7 ± 24.3  77.0 ± 25.7  42.1 ± 30.0  82.6 ± 21.7  88.5 ± 17.8  ***9199***** | 95 (90-100)  90 (70-100)  85 (65-95)  40 (15-65)  90 (75-100)  95 (85-100)  ***36206***** |
| Income | <$20,000  $20,000-$39,000  $40,000-$69,000  ≥$70,000  *F/chi-squared statistic* | 58.7 ± 32.8  77.7 ± 26.9  86.6 ± 18.7  88.3 ± 17.4  ***70.3***** | 61 (30-90)  90 (67-100)  95 (80-100)  95 (85-100)  ***214***** | 70.2 ± 29.0  82.2 ± 22.5  88.8 ± 17.1  92.6 ± 13.0  ***7428***** | 80 (50-95)  90 (75-100)  95 (85-100)  95 (90-100)  ***25713***** |
| ARIA | Major city  Inner Regional  Outer Regional  Remote/Very Remote  *F/chi-squared statistic* | 74.9 ± 29.0  75.4 ± 29.9  73.3 ± 29.7  67.4 ± 30.3  *2.41^ns^* | 89 (55-100)  90 (60-100)  85 (50-100)  70 (50-95)  ***10.6**** | 83.7 ± 23.1  83.5 ± 22.8  83.6 ± 22.9  82.6 ± 24.0  ***5.5**** | 95 (80-100)  95 (80-100)  95 (80-100)  94 (75-100)  ***60.2***** |
| SEIFA | 1 (most disadvantaged)  2  3  4  5 (least disadvantaged)  *F/chi-squared statistic* | 70.4 ± 30.6  72.4 ± 31.1  75.9 ± 29.2  79.8 ± 25.6  81.6 ± 26.0  ***6.8***** | 85 (50-100)  85 (50-100)  90 (60-100)  90 (70-100)  95 (75-100)  ***26.5***** | 80.3 ± 25.3  82.9 ± 23.3  83.2 ± 23.3  84.3 ± 22.3  87.1 ± 20.0  ***420***** | 90 (70-100)  94 (75-100)  95 (80-100)  95 (80-100)  95 (85-100)  ***1953***** |
| **BEHAVIOUAL FACTORS** | | | | | |
| Smoking status | Never smoker  Former smoker  Current smoker  *F/chi-squared statistic* | 79.4 ± 26.6  71.3 ± 31.0  69.8 ± 30.8  ***12.5***** | 90 (70-100)  85 (50-100)  80 (45-95)  ***33.5***** | 85.0 ± 22.0  82.1 ± 23.7  80.3 ± 25.4  ***414***** | 95 (80-100)  90 (75-100)  90 (70-100)  ***1580***** |
| Alcohol Consumption | 0 drinks/week  1-7 drinks/week  8+ drinks/week  *F/chi-squared statistic* | 69.2 ± 31.5  78.2 ± 27.7  79.3 ± 26.0  ***15.5***** | 80 (45-100)  90 (67-100)  90 (68-100)  ***38.6***** | 77.6 ± 27.1  86.0 ± 20.6  87.1 ± 19.2  ***2759***** | 90 (65-100)  95 (80-100)  95 (85-100)  ***5572***** |
| Body Mass Index | Underweight  Normal weight  Overweight  Obese  *F/chi-squared statistic* | 75.5 ± 26.2  79.1 ± 27.5  80.3 ± 26.2  67.4 ± 31.4  ***18.2***** | 90 (56-100)  90 (65-100)  90 (72-100)  80 (44-95)  ***71.1***** | 76.0 ± 30.2  87.2 ± 20.7  85.4 ± 20.9  76.1 ± 26.3  ***2098***** | 90 (61-100)  95 (85-100)  95 (80-100)  85 (65-95)  ***9771.9***** |
| Sufficient physical activity | No  Yes  *F/chi-squared statistic* | 65.0 ± 34.0  80.1 ± 24.4  ***50.9***** | 75 (35-95)  90 (70-100)  ***60.2***** | 75.0 ± 29.3  87.5 ± 18.1  ***8123***** | 90 (60-100)  95 (85-100)  ***8710***** |
| Sitting time | 0-3 hrs/day  4-6 hrs/day  ≥7 hrs/day  *F/chi-squared statistic* | 78.3 ± 26.4  76.2 ± 27.4  68.1 ± 33.8  ***10.5***** | 90 (61-100)  89 (60-100)  85 (40-100)  12.4* | 86.5 ± 20.1  83.8 ± 21.7  81.7 ± 25.7  ***602***** | 95 (83-100)  94 (80-100)  95 (75-100)  ***1200***** |
| Screen time | 0-3 hrs/day  4-6 hrs/day  ≥7 hrs/day  *F/chi-squared statistic* | 77.0 ± 28.2  74.0 ± 29.2  68.7 ± 32.1  ***5.6**** | 90 (61-100)  85 (55-100)  82 (40-95)  ***14.7**** | 85.7 ± 21.1  81.5 ± 23.7  83.8 ± 24.4  ***683***** | 95 (80-100)  90 (75-100)  95 (80-100)  ***2347***** |
| Vegetables intake | < 5 serves  ≥ 5 serves  *F/chi-squared statistic* | 73.8 ± 29.4  75.6 ± 29.5  ***4.0**** | 85 (55-100)  90 (55-100)  ***6.5**** | 83.7 ± 23.0  83.7 ± 22.6  ***206***** | 95 (80-100)  95 (80-100)  ***214***** |
| Fruit intake | <2 serves  ≥2 serves  *F/chi-squared statistic* | 73.5 ± 30.2  74.9 ± 29.0  *2.0 ^ns^* | 85 (55-100)  90 (56-100)  *1.8 ^ns^* | 82.6 ± 23.6  84.1 ± 22.6  ***226***** | 90 (75-100)  95 (80-100)  ***330***** |
| **PSYCHOSOCIAL FACTORS** | | | | | |
| Carer status | None  Part-time  Full-time  *F/chi-squared statistic* | 74.9 ± 29.6  72.9 ± 27.6  72.3 ± 28.8  ***2.81**** | 90 (55-100)  80 (55-95)  85 (50-100)  *7.1* | 84.0 ± 22.8  83.7 ± 21.5  75.9 ± 25.9  ***455***** | 95 (80-100)  90 (78-100)  85 (60-95)  ***1419***** |
| Self-rated health | excellent/very good  good/fair  poor  *F/chi-squared statistic* | 90.3 ± 17.0  70.1 ± 28.1  28.1 ± 26.4  ***220***** | 100 (90-100)  80 (50-95)  20 (5-40)  ***424.8***** | 92.8 ± 12.7  75.1 ± 25.3  28.8 ± 26.0  ***27189***** | 95 (90-100)  85 (60-95)  22 (5-45) ***53288***** |
| Self-rated quality of life | excellent/very good  good/fair  poor  *F/chi-squared statistic* | 88.9 ± 17.1  65.8 ± 29.9  33.0 ± 32.0  ***180***** | 95 (85-100)  75 (44-95)  25 (5-55)  ***357.5***** | 91.1 ± 14.2  72.5 ± 27.3  36.7 ± 32.7  ***20181***** | 95 (90-100)  80 (55-95)  25 (6-61)  ***38226***** |
| K10 | Low  Moderate  High  Very high  *F/chi-squared statistic* | 81.4 ± 25.2  71.4 ± 29.1  59.8 ± 32.3  46.7 ± 33.2  ***60.1***** | 94.4 (75-100)  85 (50-95)  65 (31.7-90)  40 (15-75)  ***181.2***** | 86.5 ± 20.1  77.4 ± 26.3  68.9 ± 30.4  59.1 ± 33.4  ***3986***** | 95 (85-100)  90 (65-100)  80 (45-95)  65 (30-90)  ***10412***** |
| Social contacts | None  1-3 people  4-6 people  >=7 people  *F/chi-squared statistic* | 60.6 ± 34.1  71.3 ± 30.0  77.2 ± 27.6  80.3 ± 25.9  ***16.2***** | 70 (33-90)  83 (50-100)  90 (65-100)  90 (70-100)  ***52.3***** | 79.8 ± 26.2  80.8 ± 25.2  84.2 ± 22.3  86.3 ± 20.2  ***620***** | 90 (70-100)  90 (75-100)  95 (80-100)  95 (85-100)  ***1605***** |
| Visits to family & friends/week | None  1-2 times  3-4 times  >=5 times  *F/chi-squared statistic* | 68.1 ± 32.6  75.8 ± 29.1  77.5 ± 27.5  73.6 ± 29.0  ***4.4**** | 80 (45-100)  90 (60-100)  90 (65-100)  85 (55-100)  ***13.3**** | 84.5 ± 22.4  84.7 ± 21.7  83.6 ± 22.5  77.8 ± 28.3  ***347***** | 95 (80-100)  95 (80-100)  95 (80-100)  90 (65-100)  ***767***** |
| Social group meetings/week | None  1-2 times  3-4 times  >=5 times  *F/chi-squared statistic* | 73.7 ± 30.7  75.4 ± 28.1  76.2 ± 26.0  74.0 ± 27.9  *1.3^ns^* | 89 (55-100)  89 (55-100)  85 (60- 95)  85 (50-100)  *0.9 ^ns^* | 83.1 ± 24.2  84.7 ± 21.5  83.8 ± 21.4  82.2 ± 22.9  ***104***** | 95 (80-100)  95 (80-100)  90 (80-100)  90 (75-100)  ***267***** |
| **CHRONIC DISEASE** | | | | | |
| Heart Disease | No  Yes  *F/chi-squared statistic* | 76.5 ± 28.5  57.0 ± 31.9  ***77***** | 90 (60-100)  55 (30-88)  ***67.7***** | 85.4 ± 21.5  70.0 ± 28.6  ***11100***** | 95 (80-100)  80 (50-95)  ***10960***** |
| Stroke | No  Yes  *F/chi-squared statistic* | 75.0 ± 29.2  53.6 ± 33.5  ***32***** | 90 (56-100)  55 (20-85)  ***25.9***** | 84.3 ± 22.2  60.0 ± 32.6  ***7439***** | 95 (80-100)  70 (30-90)  ***4683***** |
| Thrombosis | No  Yes  *F/chi-squared statistic* | 75.6 ± 28.9  53.1 ± 32.5  ***57***** | 90 (60-100)  53 (30-85)  ***44.6***** | 84.3 ± 22.4  68.8 ± 30.0  ***4465***** | 95 (80-100)  80 (50-95)  ***3548***** |
| High blood pressure | No  Yes  *F/chi-squared statistic* | 78.1 ± 28.1  67.8 ± 30.8  ***46***** | 90 (65-100)  80 (45-95)  ***53.9***** | 86.7 ± 20.7  77.9 ± 25.6  ***8046***** | 95 (85-100)  90 (67-95)  ***10462***** |
| Diabetes | No  Yes  *F/chi-squared statistic* | 77.6 ± 27.7  57.1 ± 32.8  ***113***** | 90 (65-100)  60 (30-85)  ***98.6***** | 84.8 ± 21.9  70.6 ± 29.4  ***7095***** | 95 (80-100)  80 (50-95)  ***5830***** |
| Asthma/  Hayfever | No  Yes  *F/chi-squared statistic* | 77.0 ± 28.4  67.0 ± 31.4  ***38***** | 90 (60-100)  75 (45-95)  ***43.4***** | 84.4 ± 22.5  80.6 ± 25.5  ***1051***** | 95 (80-100)  90 (75-100)  ***1401***** |
| Depression/  Anxiety* | No  Yes  *F/chi-squared statistic* | 79.5 ± 26.3  63.4 ± 33.0  ***92***** | 90 (67-100)  75 (35-95)  ***79.3***** | 85.1 ± 21.7  77.2 ± 26.7  ***3730***** | 95 (80-100)  90 (65-100)  ***3273***** |
| Cancer ** | No  Yes  *F/chi-squared statistic* | 75.1 ± 29.2  66.3 ± 31.4  ***14**** | 90 (60-100)  80 (39-94)  ***17.5***** | 84.6 ± 22.3  76.0 ± 26.6  ***3210***** | 95 (80-100)  85 (65-95)  ***3673***** |
| None | No  Yes  *F/chi-squared statistic* | 71.4 ± 30.5  87.5 ± 20.6  ***69***** | 85 (50-95)  95 (85-100)  ***81.5***** | 81.6 ± 24.0  91.5 ± 16.0  ***3484***** | 90 (75-100)  100 (90-100)  ***9971***** |
| Number of conditions | 0  1-2  3-4  5-6  7 or more  *F/chi-squared statistic* | 87.5 ± 20.9  76.6 ± 27.5  59.5 ± 31.8  47.0 ± 32.2  44.4 ± 32.0  ***72***** | 95 (85-100)  90 (60-100)  65 (30-90)  44 (20-75)  40 (15-65)  ***245.3***** | 91.0 ± 16.6  84.9 ± 21.2  72.9 ± 27.2  59.2 ± 30.5  53.8 ± 32.8  ***7013***** | 100 (90-100)  95 (80-100)  85 (60-95)  65 (35-85)  55 (25-85)  ***26589***** |

*P<0.05; **P<0.0001; ns=non-significant
